# Vocal health of parents of children with hearing assistive devices

**DOI:** 10.12669/pjms.39.5.7570

**Published:** 2023

**Authors:** Uzma Qayyum, Nazia Mumtaz, Ghulam Saqulain

**Affiliations:** 1 Uzma Qayyum, MS (SLP) Speech Language Pathologist, Department of Speech Language Pathology, Riphah International University, Lahore, Pakistan; 2 Nazia Mumtaz, FCPS (Rehab Sciences) Head of Department, Department of Speech Language Pathology, Faculty of Rehab and Allied Health Sciences, Riphah International University, Lahore, Pakistan; 3 Ghulam Saqulain, FCPS (Otorhinolaryngology) Head of Department & Professor, Department of Otorhinolaryngology, Capital Hospital PGMI, Islamabad, Pakistan

**Keywords:** Vocal health, Voice related quality of life, Voice handicap index, Hearing loss, Hearing impaired Children, Hearing assistive devices

## Abstract

**Background & Objectives:**

VH (Vocal health) is the need of the hour. VH of parents of children with hearing assistive devices (HAD) reveals a literature gap, during the habilitation process of their children. To explore the vocal health of parents of children with hearing assistive devices.

**Methods:**

This cross-sectional study was conducted at Riphah International University from September to December 2021. Study recruited N=384 parents of Hearing Impaired children (HIC) using HAD for at least two years, of both genders and aged 2-9 years using convenience sampling. Voice-related quality of life (V-RQOL), and vocal health Index (VHI) -10 were used for data collection. Data was analyzed on SPSS Version 25. Descriptive statistics, Anova and t-test were utilized to see difference between means of groups. P<0.05 shows significant-results.

**Results:**

Parents of children using hearing assistive devices had excellent V-RQOL score in 350(91.14%) parents. There was no significant difference in V=RQOL as regards type of hearing assistive device use (p=0.102), laterality of device use (p=0.918) and degree of hearing loss (p=0.143). However, type of hearing loss revealed significant difference (p=0.021). Also VHI score revealed significantly (p=0.008) lower means in parents of children with cochlear implants.

**Conclusion:**

Current study concludes that the parents raising hearing impaired children with hearing assistive devices, possess good vocal health as determined by VHI and V-RQOL scores with only a very small number of parents reporting vocal symptoms.

## INTRODUCTION

Voice is essential for communication in daily life and it delivers required information including emotions, individuals’ traits and social status. Voice production is the end result of complicated fluid-structure-acoustic interactive sequences depending on structural properties of lungs, voice box and airway. The resultant outcome of this being voice acoustics and perception with imposition of variations due to vocal physiology and physical principles on the intensity, quality, frequency or pitch, prosody and overall modulation of voice. These characteristics ultimately convey meaning^1^ and identity in voice^2^, including non-verbal alterations in voice to express emotions which are rapidly and accurately understood.^3^

Voice is used to pass on perceptual sensations with humans having an oblivious relationship with ability to interpret depictions of voice quality. However, this is not valid for some instrumental estimations of voice quality, hence self-assessment tools are used detect the different aspect related to vocal functioning which is not possible with instrumental estimations like acoustic assessments.^4^ When the voice quality, intensity and pitch or clamor become altered or is not appropriate for a person’s age, culture, gender and geographic region it is labelled as a voice disorder as defined by American Speech Language-Hearing Association.^5^ It can be of structural, neurogenic or functional varieties.^5^

A study involving teachers of different classes and subjects reveal different level of vocal issues with a higher frequency of vocal abnormalities in primary teachers compared to instructors of optional subjects.^6^ Deleterious effects of overuse and misuse of voice do occur even in student Speech Language Pathologists (SLP) and the difference is evident even after voice use.^7^ Hearing impairment is a common disability with a high prevalence of 14.27 % reported from Iran being a neighboring country with 9.52% having grade 1, 4.04 Grade-2, 0.67 Grade-3 and 0.48 having total deafness.^8^ With 90 percent of deaf infants born to hearing parents vocal communication is even more challenging.^9^

A local study noted that around 1.6 out of 1000 individuals suffer profound hearing impairment involving both ears.^10^ Mumtaz N et al. directed an investigation which noted late detection of hearing impairment in Pakistan,^11^ and a prevalence of hearing aid use of 32% in cases with hearing loss (HL) was noted in another study.^12^ Hence for these families, having a young person with hearing-impairment with late detection and inability to benefit from HA is a devastating situation.

The advancement of hearing-related innovations has been working on step by step, for example, cochlear implants (CI) have compressed the impact of deafness by working with children with HI.^13^ A review by Blank A. et al. revealed that the parents of children wearing hearing devices experience explicit administration issues, the greater the stress of parents in families with HI children, the lesser would be the interaction between the parent and child and turn-taking or conversation between child and parent are adversely affected.

The stressful conditions make the parents less responsive, more controlling and this ultimately leads to low quality and quantity of verbal communication.^14^ According to Whistling S et al. parents and caregivers of even typically hearing children have to talk more and in varying situations need to repeat or rephrase their statements to benefit their children thus engaging parents into vocal loading tasks which is ultimately injurious for their vocal health.^15^ Hence current study was conducted with the objective to explore the vocal health of parents of children with hearing assistive devices. Findings of the study may help effective planning of preventive interventions, help clinicians better manage and act as a research base for future studies.

## METHODS

This cross-sectional study was conducted at Riphah International University over a period of 16 weeks 1st September 2021 to 31^st^ December, 2021. Study recruited N=384 parents of hearing impaired children using convenience sampling from Pakistan Air Force Special Children’s School, Inayat Foundation, Alam School of Speech & Learning, Rangers Institute of Special Children and mainstream section of Hamza Foundation, Lahore. Sample included parents of HI children of both genders aged 2-9 years using hearing assistive devices for at least two years. Parents with poor health, known cases of asthma, nodules or polyps, on job with any kind of vocal stress and parents of children with any comorbid condition or any additional disability like cerebral palsy, seizures, visual problems, Downs syndrome etc., were excluded from the study. Sample size was calculated 384 on the basis of total population of hearing-impaired children 200,000 approximately by using 95% confidence level and 5% margin of error through Rao-soft online sample size calculator. Study was initiated following ethical approval of research from Research Ethics Committee of Riphah College of Rehab & Allied Health Sciences, Riphah International University vide registration No RCR-RE-MS-SLP/Spring 21/004 and informed consent of participants.

Basic demographic sheet, voice-related quality of life (V-RQOL),^16^ and Urdu version -10 of vocal health Index (VHI)^17^ were used for data collection. Urdu version VHI-10 is a valid tool and consists of 10 questions in total which can be further divided into three domains functional, emotional, and physical aspects. V-RQOL^16^ is a valid and reliable 10-item tool as reported by Hogikyan & Sethuraman, scoring algorithm for VR-QOL measures, social-emotional domain and physical-functional domain V-RQOL General Scoring Algorithm.

100 - (Raw Score - # items in domain or total) x 100

(Highest Possible Raw Score - # items)

SPSS Version 25 was utilized for data analysis. Descriptive statistics were used with frequency and percentage calculated for categorical variables and chi-square used to see any difference between groups of child hearing characteristics, while Anova and t-test were utilized to see difference between means of groups. P<0.05 was considered significant.

## RESULTS

**Fig.1:**
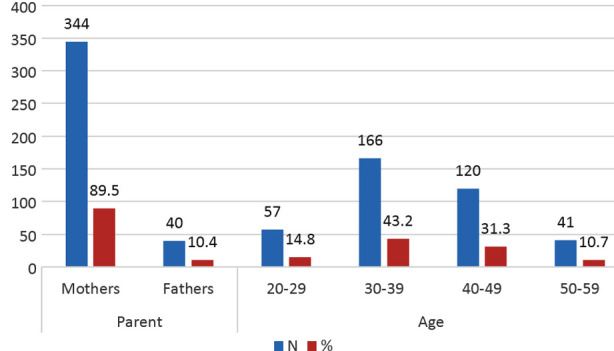
Age and gender distribution (n=384).

Study sample comprised mainly 344(89.6%) mothers of HI children using hearing assistive devices with most 166(43.2%) aged 30-39 years (Fig.1). Current study revealed that parents of HI children using hearing assistive devices had excellent V-RQOL score in 350(91.14%) parents and only 6(1.6%) had poor V-RQOL score (Table-I). Also, no parent in the cochlear implanted category had poor score, however as regards type of hearing assistive device use did not reveal any significant difference in V-RQOL with p=0.102. Most children 259(67.4%) were using bilateral hearing devices, however laterality in terms of use of unilateral or bilateral hearing assistive device also did not reveal significant difference with p=0.918. There was significant (p=0.021) difference in V-RQOL scores as regards type of hearing loss with mixed hearing loss having more cases with poor V-RQOL scores. Degree of HL did not reveal any significant difference (p=0.143) in V-RQOL scores.

**Table-I:** Child hearing characteristics Versus V-RQOL Scale Category Cross Tabulation (n=384).

Variable	Group	Frequency of VR-QOL Scale Category				

		10-15(Excellent) [350(91.14)]	16-20(Very Good) [16(4.17)]	21-25(Good) [10(2.60)]	26-30(Fair) [2(0.52)]	>30(Poor) [6(1.6)]
Type of Hearing Assistive Device	Cochlear Implant [111(28.9)]	107	1	2	1	0
	Hearing Aid [273(71.1)]	243	15	8	1	6
	X2, P-value	7.727, 0.102				
Laterality of Hearing Assistive Device	Unilaterally [125[32.6)]	114	4	4	1	2
	Bilaterally [259(67.4)]	236	12	6	1	4
	X2, P-value	0.947, 0.918				
Type of Hearing Loss	Conductive [92(24)]	81	5	5	1	0
	Sensory-Neural [220(57.3)]	208	6	3	1	2
	Mixed Hearing Loss [72(18.8)]	61	5	2	0	4
	X2, P-value	17.969, 0.021				
Degree of hearing loss of the child	Mild [73(19)]	61	8	3	1	0
	Moderate [99(25.8)]	93	1	3	0	2
	Severe [94(24.5)]	85	4	2	1	2
	Profound [118(30.7)]	111	3	2	0	2
	X2, P-value	17.173, 0.143				

Study found marked difference in the VHI mean score when compared with V-RQOL score categories with lowest score (1.84±1.349) noted in the category of lowest V-RQOL i.e., Excellent (10-15) and difference was significant (p=0.000) when compared with other categories. Almost similar results were noted in the subscale of Functional, Emotional and Physical subscales (p<0.000) with lowest scores in the Excellent category of V-RQOL, (Table-II).

**Table-II:** Comparison of VHI Scale & Subscale mean score with V-RQOL score category: Anova Statistics (N=384).

Scale/ Subscale	Result Category V-RQOL	N	Mean±SD	F	P-value
Functional Subscale of VHI-10 (Urdu)	10-15(Excellent)	350	0.8±1.649	160.78	0.000
	16-20(Very Good)	16	5.38±2.363		
	21-25(Good)	10	6.8±2.781		
	26-30(Fair)	2	10±0		
	>30(Poor)	6	14.83±1.835		
	Total	384	1.42±2.808		
Emotional Subscale of VHI-10 (Urdu)	10-15(Excellent)	350	0.05±0.318	243.25	0.000
	16-20(Very Good)	16	1.19±2.007		
	21-25(Good)	10	1.9±1.101		
	26-30(Fair)	2	2±0		
	>30(Poor)	6	6.83±1.941		
	Total	384	0.26±1.081		
Physical Subscale of VHI-10 (Urdu)	10-15(Excellent)	350	0.37±0.797	186.84	0.000
	16-20(Very Good)	16	3.13±1.821		
	21-25(Good)	10	4.2±1.549		
	26-30(Fair)	2	3±0		
	>30(Poor)	6	9.67±0.816		
	Total	384	0.74±1.651		
Total Score of VHI	10-15(Excellent)	350	1.84±1.349	21.57	0.000
	16-20(Very Good)	16	4±0		
	21-25(Good)	10	4±0		
	26-30(Fair)	2	4±0		
	>30(Poor)	6	4±0		
	Total	384	2.03±1.42		

VHI-10 total scale and subscale mean scores revealed lower means in parents of children using cochlear implant compared to hearing aids and the difference was significant for total mean score (p=0.008), functional subscale (p=0.011) & physical subscale (p=0.016), however it was not significant for emotional subscale (p=0.104) (Table-III). Interestingly V-RQOL total and standard scores (p=.011) for SED (p=.035) and PFD (p=.012) were significantly lower in parents of children using hearing aids compared to cochlear implants and difference was significant.

**Table-III:** VHI-10 & V-RQOL scale and subscale mean scores versus Hearing Assistive Devices Cross Tabulation: T-Test statistics.

Scale	Subscale	Score With Hearing Assistive Device Being Used by the Child		T-Test Statistics

		Cochlear Implant (n=111) Mean±SD	Hearing Aid (n=273) Mean±SD	T, p-Value
VHI-10 (Urdu)	Functional Subscale	0.85±1.908	1.65±3.073	-2.554, .011
	Emotional Subscale	0.12±0.552	0.32±1.229	-1.630, .104
	Physical Subscale	0.42±0.996	0.87±1.838	-2.428,.016
	Total Score	1.73±1.293	2.15±1.462	-2.662, .008
VR-QOL	Standard Score SED	99.04±5.343	96.27±13.343	2.121,.035
	Standard Score PFD	95.31±8.615	91.96±12.852	2.525,.012
	Standard Total Score	96.8±6.633	93.68±12.123	2.559,.011

## DISCUSSION

Study measured subjective outcomes of voice related quality of life by using V-RQOL measure and VHI, using affected parents’ perspectives regarding various aspects of their vocal loading, voice fatigue, vocal abuse or misuse of voice related quality of life. With a sample which comprised predominantly 344(89.6%) mothers & 40(10.4%) fathers of HI children using hearing assistive devices with majority 166(43.2%) aged 30-39 years, study revealed that parents of HI children using hearing assistive devices had excellent V-RQOL score in majority 350(91.14%) parents and only 6(1.6%) had poor V-RQOL score. Also, V-RQOL did not reveal significant association with degree of HL (p=0.143) and laterality of hearing assistive device use (p=0.918), though there was significant association (p=0.021) with type of HL with mixed HL having more cases with poor V-RQOL. This is in compliance with literature where in a local study by Ishtiaq N et al revealed low level of stress in parents of HI children compared to parents of children with autism.^18^ Similarly Blank A et al. reported negative correlation with child’s language comprehension and parenting stress and QoL.^19^

In present study type of hearing assistive device use did not reveal any significant difference (p=0.102) with V-RQOL, though there was no parent in cochlear implanted category having poor score. This is in conformity with a study by Wischmann S et al. in 2022, aimed to investigate long-term development of basic language in new generation of children with hearing-impairment (HI) using hearing aids (HA) for hearing loss of 30dB to 80dB and bilateral CI for hearing loss more than 80 dB, revealed normal social wellbeing and language skills, which favors the fact that parental vocal health and quality of life is not affected.^20^ In contrast a local study noted increased stress in parents of children with CI compared to those with normal hearing as well as those using Has.^21^ Similarly, a study by Chen CH et al. revealed that naming utterances and counts of sustained attention were same in HL children with amplification and without amplification, however those without amplification revealed less synchronization of these events.^22^

Morningstar M et al. in their study reported that Interaction between infant and parent can alter parental prosody, which is good for infants’ language development.^23^ However, children with HAs and CIs can benefit from of parental language input.^24^ In current study VHI-10 total scale and subscale mean scores revealed lower means in parents of children using CI compared to HAs and the difference was significant for total mean score (p=0.008), functional subscale (p=0.011) and physical subscale (p=0.016), however it was not significant for emotional subscale (p=0.104). Interestingly V-RQOL total and standard scores (p=0.011) for SED (p=0.035) and PFD (p=0.012) were significantly lower in parents of children using HAs compared to CI and difference was significant. This may be due to the fact that parents of kids using CI face various needs and also face problems depending upon their condition and experience.^25^

Current study found marked difference in the VHI mean score when compared with V-RQOL score categories with lowest score (1.84±1.349) noted in the category of lowest V-RQOL ie. Excellent (10-15) and difference was significant (p=0.000) when compared with other categories. Almost similar results were noted in the subscale of Functional, Emotional and Physical subscales (p<0.000) with lowest scores in the Excellent category of V-RQOL. A moderate to strong association between VHI and its domains with V-RQOL have been reported by Lu D et al. in a study involving teachers.^26^

### Limitations:

Voice related Quality of life (V-RQOL) was administered in English language which is not the mother tongue of the sample population which could induce biasness in scores.

## CONCLUSIONS

Current study concludes that the parents raising hearing impaired children with hearing assistive devices, possess good vocal health as determined by VHI and V-RQOL scores with only a very small number of parents reporting vocal symptoms.

### Authors Contribution:

**UQ:** Did the data collection, analysis and interpretation and responsible for integrity of the research.

**NM:** Did the supervision and critical revision of the article

**GS:** Did the conception of work and writing of the manuscript.
